# Influencing factors on cervical cancer screening from the Kurdish women’s perspective: A qualitative study

**Published:** 2015

**Authors:** VH Rasul, MA Cheraghi, Z Behboodi Moqadam

**Affiliations:** * Faculty of Nursing and Midwifery, International Campus, Tehran University of Medical Sciences, Tehran, Iran; Shaqlawa Techniqueal Institute, Erbil Polytechnique University, Kurdistan Region, Iraq; ** Faculty of Nursing and Midwifery, International Campus, Tehran University of Medical Sciences, Tehran, Iran; *** Reproductive Health, Faculty of Nursing and Midwifery, International Campus, Tehran University of Medical Sciences, Tehran, Iran

**Keywords:** cervical cancer screening, Kurdish women, content analysis, qualitative research

## Abstract

**Aim:**This study was aimed to explore and describe the Kurdish women’s perception of cervical cancer screening.

**Methods:** A qualitative design based on a conventional content analysis approach. Purposive sampling was applied to 19 women chosen, who had a Pap smear or refused to have one. The study was performed in the Kurdistan Region, Iraq. Semi-structure din-depth individual interviews were carried out to collect data.

**Results: **Four main themes including conflict, belief, and awareness about cervical cancer screening and socio-cultural factors emerged during data analysis

**Conclusions: **Cervical cancer has a high mortality rate in the developing countries. However, only a few Kurdish women participated in the cervical cancer screening in the Kurdistan Region, Iraq. Understanding the factors associated with the women’s perception of cervical cancer could guide future educational planning and clinical interventions improve the cervical cancer screening.

## Introduction

Cervical cancer is a major health problem and the second leading cause of cancer deaths among women worldwide. While about 500,000 women develop cervical cancer per annum, the survival rate is as low as 50% [**1**,**2**]. In addition, a great majority (over 86%) of the new cases of cervical cancer are reported from developing countries [**3**,**4**]. The primary aim of cervical cancer screening is to decrease the incidence of invasive cervical cancer by the early detection and treatment of the precursors of the cancer. The secondary aim is to reduce the mortality by the timely detection of the invasive cancers [**5**].

According to the World Health Organization (WHO), the crude incidence rate of cervical cancer in Iraq is 2.1 per 100,000 women of all ages. Moreover, 10.21 million Iraqi women aged 15 years and older are at risk of developing the disease[**6**].

The Kurdistan Region is an autonomous region in Northern Iraq. It covers an area of about 40,000 square kilometers and holds 8.35 million people (from 36 million people living in Iraq) [**7**].

Although some independent reports from different cities of Iraq have shown an increased incidence of different types of cancer, limited research has evaluated the cancer incidence in the country, especially in the Kurdistan Region. In the first study on cancer incidence in the Kurdistan Region, Ramadhan et al. (2011) reported evidence of an increased risk of all cancers, including cervical cancer, in recent years [**8**]. Exposure to numerous environmental and epidemiological changes in the Kurdistan Region of Iraq has elevated the risk of cancer in this region. For instance, due to the persistent effects of the chemical bombardment of Halabja City, Kurdistan, in 1988, the incidence of cancer in this city is 10 times higher than the normal rate [**9**]. 

According to the WHO scanning, even at every 10 years, can decrease the incidence of cervical cancer by 64% [**10**]. The idea behind the PAP-test is that cellular changes that may develop into cancer are detected at such an early stage that they can be removed through a simple operation, thus preventing the cancer [**11**]. The natural history of an invasive CC, a disease with long preneoplastic changes, more than 10 years in the majority of the cases, generally allows its early detection [**12**]. The survival rate of cervical cancer is directly related to the stage of diagnosis, i.e. patients with an early diagnosis have a significantly higher survival rate than those suffering from metastatic disease (91% vs. 14%]) [**13**,**14**].

No screening programs for cervical cancer existed in the Kurdistan Region until 2004. The first study on cervical intraepithelial neoplasia (CIN) in the Kurdistan Region was performed by the Ministry of Health. Since the results indicated the presence of dysplasia in 4.4% of the population, the Ministry of Health established a cervical cancer screening program in the Kurdistan Region. The program was also initiated in Erbil in 2006, in Duhok in 2008, and in Sulemani in 2009 [**15**]. However, the WHO reported an absence of data about the estimated coverage of cervical cancer screening in Iraq by age and study [**6**]. While there is no specific obligation to perform cervical cancer screening, the physicians’ recommendation seems to be the most important motivator in this regard. Since little knowledge is available about the determinants of Kurdish women’s participation in screening programs, enhancing the overall cancer screening rates will widely depend on the information about the factors associated with screening for various cancer types including cervical cancer. As no qualitative study has focused on this subject in the Kurdistan Region, the present research sought to describe and explore the experiences of Kurdish women of cervical cancer screening.

## Methods

**Design**

A qualitative design based on a conventional content analysis approach was used. Qualitative research is a form of social inquiry that focuses on the way people make sense of their experiences and the world in which they live [**16**]. Content analysis is potentially one of the most important research techniques in the social sciences that the researcher examines artifacts of social communication [**17**].

**Objectives**

The current study aimed to explore the perspectives of the participants about the influencing factors on cervical cancer screening programs.

**Data collection and participants**

Semi-structured in-depth interviews were performed with 19 women who either had a Pap test or refused to take the test in the Kurdistan Region, Iraq. The participants should have been chosen based on purposeful sampling strategy according to the maximum variation technique. The criteria for the samples inclusion for this study were: a) Married women who agreed to participate in the study, b) Kurdish women who lived in the Kurdistan Region, Iraq, c) The women who had experience regarding cervical cancer screening (referred or not referred) in a governmental or private section, d) The women who had a health checkup profile in the healthcare centers, who did not follow a cervical cancer screening program. Non Iraqi Kurdish women were excluded. Recruitment continued as long as new themes continued emerging from the interviews, and data saturation was reached at 16 interviews and the 17th,18th,19th did not have any extra information.

The interviews were individually conducted in the Pap smear center andthe participants'workplace. Each interview lasted about 40-72 minutes. Data were collected from May 14 to November 20, 2014. The individual interview questions were:

• What is the women’s perception regarding cervical cancer

• What is the meaning of cervical cancer screening from their perspective

• Have you ever had a cervical cancer screening test? If yes, what would you say about your experience? If you have never received a cervical cancer screening test, could you sharethe reasons?

The interviews were conducted in the participants’ native language (Kurdish) and then translated into English by first author as an emic researcher. A bilingual speaker then revised and confirmed the translations. Each interview was recorded and transcribed verbatim and then analyzed concurrently [**18**,**19**].

**Ethical considerations**

The Ethics Committee of Tehran University of Medical Sciences (Project Number: 130/ 1117, Approval Date: 10/08/2014) and Ministry of Health and Director of Health in Erbil (Number: 65, Date: 12/05/2014) approved the study proposal and confirmed its ethical considerations.All participants were informed and explained the reasons and the methods of the study. Individuals who agreed to participate in the study signed written consent forms and allowed the tape-recording of the interviews. They were also ensured about the voluntary nature of participation and their right to withdraw at any time.

**Data analysis**

Based on the instructions of Graneheim and Landman, the interviews were transcribed verbatim and the transcriptions were read thoroughly several times [**18**]. After acquiring a general idea of the subject, the obtained texts were broken into condensed meaning units. These meaning units were abstracted and coded. The codes were then compared and allocated to different subcategories and categories based on their similarities and differences. A number of themes were ultimately formulated to express the latent content of the text.

**Trustworthiness**

Member checking, peer checking, and prolonged engagement were present to guarantee trustworthiness [**19**]. In order to perform member checking, the participating women were asked to compare the results with their experiences and verify the findings. Peer checking, to enhance the accuracy and trustworthiness of the findings, was conducted by two expert supervisors (and faculty members). The researchers independently analyzed the data by identifying and categorizing codes from the subjects’ responses to each question. The two teachers (C. M. and B. Z.) thenreviewed the transcriptions and the extracted codes. In areas where the two did not agree, definitions were clarified and discussion was continued until consensus was reached. In an attempt to ensure a prolonged engagement, one of the researchers (a PhD candidate of nursing; R. V. H.) attended the research field and tried to get the participants’ trust and collect in-depth data.

**Findings**

**General characteristics of the participants**

The mean age of the subjects was 39.2 years (range: 25-52 years). The age of married participants was 14-28 years. While some interviewees were illiterate, some held a doctorate degree. Half of the subjects were employed. About 89.4% of the respondents were married, one (5.3) was divorced, and one (5.3) was widowed. Moreover, half of the studied women lived in urban and others in rural areas. 

**Themes**

Four main themes including conflict, belief, and awarenessabout cervical cancer screening and socio-cultural factors emerged during the data analysis. Categories, subcategories are represented in **Table 1** .

**Table 1:** Main categories and relative sub-categories

No	Categories	sub-categories
1	conflict	existence of fear
		health seeking behaviors
		willing and able to decision-making
2	belief	health-related beliefs
		cervical cancer screening-related beliefs
3	awareness about cervical cancer screening	appropriate awareness
		inappropriate awareness
		lack of awareness
4	socio-cultural factors	supportive family
		spirituality
		role of mass media
		influence of other women
		influence of physicians and healthcare providers

**Conflict**

The conflict was the main theme, which was repeatedly mentioned in the statements of the participants. It described a continuous mental involvement regarding the decision whether to take the screening test or not. Such a self-conflict was present not only before the test, but also after it (before receiving the results).

One of the participants declared: “Can you believe that I had to go receive my test results yesterday, but I was hesitant? At first, I thought to myself that I had to trust in God. I decided I did not want to know if I was sick or if I was suffering from a disease. I wanted to leave it to destiny. However, after that, I said no and I convinced myself that if I were ill, the sooner I knew, the better it would be for me. I thought this would help me leave this fear and this situation sooner and I might cure myself for the rest and trust in God.”

This category had three sub-categories (Health seeking behaviors, Existence of Fear and Willing and able to decision-making). In this study, emotional factors were identified among women as the most important ones for taking the Pap test. Women who were interested in their health said that if every test was essential for their health, they would do it.

Existence of Fear generally arose from pain, unfamiliarity with the test (its purpose and procedure) and a positive or abnormal result and treatment in case of cancer (expensive and side effects of drugs for the treatment of cancer were applied) was also common among the participants. Death was the first thing that came to the minds of most participants when they heard the word.

Another participant who had never been screened disclosed that: “I thought about it [Pap smear] many times, but have I decided to go and do the test? No, notuntil now!Because my fearis becoming greater and this fear did not allow me to take this decision for doing the test. ”

Making a decision is an important step for cervical cancer screening. On the one hand, participants seek health behavior and understand the necessity of doing the test for women. This makes them willing and able to make the decision. On the other hand, the fear of test pain and the possibility of a positive result lead to a self-conflict, which may eventually make the person refuse the test.

**Belief**

Another main theme extracted from the participants’ statements was belief. It focused on personal mental representation and perceptions about health and the necessity of disease prevention and screening. This theme consisted of two sub-themes including health-related beliefs and cervical cancer screening-related beliefs. Beliefs area major element in decision-making. As expected, the participants had health-related beliefs that affected their preventive behavior. The subjects expressed the significance of their own health and believed that screening had an important effect on their health. A participant mentioned: “Everyone is always looking for their own ways of protecting their health and preventing diseases. So, if you're suffering from any disease, you can treat it.”

Cervical cancer screening sub-theme included correct and incorrect beliefs related to Pap test. Some participants who believed that all women were at risk of developing cervical cancer tended to have a Pap test. As one informant said: “Pap test is used for the early diagnosis of cervical cancer. All women should have this test done three months after their marriage. If the result is normal, the test should be repeated every three years afterward.”

Meanwhile, some participants had incorrect beliefs related to the need for a Pap test. For instance, they believed that those who had gynecological problems were at risk of cancer. Some others believed that just old women needed to have a Pap test or that trust in God would protect them from developing cancer. Obviously, those who perceived a Pap test to have a low necessity did not feel the need for screening. A woman explained that: “In my opinion, a Pap test is necessary for those who are married or suffering from genital infections or those who have a problem and an abnormal condition. I have had none of these cases, you know, I have been married for 15 years and even eight months after separating from my husband, I had no problems. Therefore, I do not think I need a Pap smear.”

**Awareness about cervical cancer screening**

This theme indicated the participants’ information and understanding about cervical cancer screening. It consisted of three sub-themes, namely appropriate awareness, inappropriate awareness, and lack of awareness.

Appropriate awareness covered the women’s correct information obtained from appropriate sources such as health care providers, books, and mass media. One of the participants explained: “Because I was very young when I got married and there are sores in my cervix, my doctor told me to take care of myself. She told me that I might suffer from cervical cancer in the future and that this test [Pap test] would diagnose the disease early.”

Inappropriate awareness indicated the participants’ misinformation obtained from invalid sources like family, friends, or other women. A woman who lived in the rural area and whose husband died from cancer stated: “I consulted with some of the women in my neighborhood and they had the same idea and they said that maybe I got the disease from my husband.”

Lack of awareness included the gaps in knowledge identified by the participants. The subjects commonly lacked knowledge about what a Pap test was, which cancer it was used for, and how often one it should be done, and why it was performed. One of the informants said: “I do not know anything about when I should start [Pap test].”

Another woman who was screened following her doctor’s recommendation declared: “Because I did not know what this test was useful for, I thought that it was an important test for diagnosing my disease.”

**Socio-cultural factors**

The participants’ statements about factors affecting their health behaviors through the larger environmental system were categorized in a theme called socio-cultural factors. 

This theme highlighted the importance of social relationships in one’s life and comprised five sub-themes, namely supportive family, spirituality, role ofmass media, influence of other women, and influence of physicians and healthcare providers.

The first sub-theme was family related to the role of a family in a woman’s health. Those women who had children responded that their children were the most important aspect of their life. The women mentioned that their health was important for the children's future. At the same time, the participants who expressed those families had a supportive role in getting Pap test. This sub-theme included the family support, encouragement, and companionship for doing the test. As a participant stated: “My family encouraged me a lot;evenmy fathertold me he would help me ifI needed anything.”

The second sub-theme (spirituality) covered behaviors that rooted in religion. These behaviors included praying, trust inGod, and belief in destiny. One participant with a gynecological problem who had delayed her Pap test until the doctor’s recommendation stated: “I always say in God we trust! With this hope, I consider myself to be away from this disease and I said Insha’Allah God will protect me from this disease. For this reason, I have not been thinking about the test so much.”

The third sub-theme, i.e. the role ofmass media, emphasized the significance of media in information provision. Mass media, e.g. television, radio, newspapers, and magazines, play a crucial role in the construction of the public beliefs about health. They can simultaneously convey the same massage to large numbers of people. One of the participants expressed that: “Because this subject is repeated in neither the community nor the mass media such as television or newspapers, perhaps no one can remember this. The media does not pay attention to this matter. In Kurdistan, very little is spoken about this topic.”

The forth sub-theme in this theme, i.e. the influence of women’s attitudes/ behaviors, highlighted the influence of others and peers on the participants’ health and screening knowledge acquisition and tendency to have a Pap test. Some women justified their lack of interest in having a Pap test by the fact that none of their family members or women around them had taken the test. A participant stated: “If I saw that most otherwomen took thetest, it would possiblyinfluence me. I would say that the test would not kill anyone and that everyonecoulddothis test. So, why shouldn’t I do it? This could make meto overcome my fear little by little to go and do the test.”

Similarly, the influence of physicians and healthcare providers on the participants’ cervical cancer screening behaviors was underscored by most participants. Some women indicated that their doctors played a role in their Pap test knowledge acquisition and the decision to have a Pap smear. Some subjects also reported that they did not have a Pap test because their doctor did not explain the necessity of the test or did not remind them to have one. For example, a participant who did not recently have a Pap test declared that she would have done a Pap test if her doctor had recommended her to do so: “Our health care system does not push us or force us to go and have a Pap smear. For instance, if I have a gynecology problem,I go to a gynecologist. However, my doctor never asks if I have had a checkup. She never tells me that it is good to do this test. She would never ask this question to encourage us to come and have the test.”

## Discussion

The present study sought to explore the women’s perspectives of cervical cancer screening in the Kurdish culture and context. Based on our findings, cervical cancer screening is a process that starts with a woman’s decision to have the test. Moreover, all our participants were able and free to make decisions. We also found that women had to deal with a series of conflicts and worries while making a decision to have a Pap test. Furthermore, women who were or were not screened had to cope with different worries and fears. In addition to their fear of the test itself (e.g. pain and unknown nature), they were worried about having cancer. Almost all participating women associated cancer with death. Some of them described cancer as a death sentence without a cure and thus evaded the test to avoid a positive result. In contrast, the screeners in this study emphasized the significance of a Pap test in preventing death due to cancer. Similar findings were reported by Guilfoyle et al. [**20**] and Hatcher et al. [**21**]. However, fear and anxiety about having a Pap test was only reported by one-third of the participants in a study in England. Moreover, women who had at least once attended the cervical cancer screening were less likely to express fear and anxiety [**22**]. Since the mentioned conflicts exerted significant effects on women’s tendency to participate in cervical cancer screening, providing clear information about a Pap test is essential to increase the willingness to have the test.

According to the Kurdish women in the current research, beliefs and knowledge about cervical cancer screening played a key role in the decision to have a Pap test. Women were more likely to have the test if there were prevailing beliefs about the risk of getting cancer such as having symptoms. However, women who believed they were at a lower risk of getting cancer or those who had no symptoms were less willing to have the test. Likewise, trusting in God and belief in destiny reduced the chances of a woman having a Pap test and this was one of the reasons for refusing the test.

Previous research has also highlighted the relation between low perceived risk of cervical cancer and low uptake of screening [**21**,**23**-**25**]. Therefore, the women’s awareness about their risk of cervical cancer has to be enhanced to increase their participation in screening programs. Likewise, trusting in God and belief in destiny reduced the chances of a woman having a Pap test and this was one of the reasons for refusing the test. For instance, they believed that a Pap test was only needed when they had some symptoms or when their doctor recommended it. Wong et al. [**26**], Al-Naggar and Isa [**27**] and Ma et al. [**24**] reported similar findings.

The results of the present study revealed that all health decisions were made or rationalized based on awareness. In fact, awareness about a Pap test, its purpose, importance, technique, procedure, advantages, and disadvantages can affect a woman’s decision to participate in cervical cancer screening. Women with less knowledge on the importance and purpose of Pap tests had a lower chance of having the test. Considering the limited knowledge of some participants, increasing education about the cervical cancer and Pap smearwas necessary to improve Kurdish women’s understanding and perception of these subjects [**25**,**28**-**30**]. Therefore, the women’s awareness about their risk of cervical cancer has to be enhanced to increase their participation in screening programs.

According to our findings, increased knowledge about cervical cancer and the Pap test led to a positive perception regarding the health promotion and disease prevention and created an accurate base for correct beliefs about the Pap test. The screeners and other participants in the present research reported that the Kurdish women had limited awareness and misconceptions about cervical cancer, the Pap test, and women’s health issues. For instance, they believed that a Pap test was only needed when they had some symptoms or when their doctor recommended it. Wong et al. [**26**], Al-Naggar and Isa [**27**] and Ma et al. [**24**] reported similar findings.

Before having a Pap test, a woman has to accept the test, i.e. women’s impression of the test can largely affect their tendency of having or not having the test. Some participants discussed their embarrassment, fear of being unfamiliar with the test and pain of test causing the test refusal. However, others said that they have taken the test with these negative feelings.Consistent findings were also suggested by previous research [**27**,**31**,**32**].

Women who had undergone a Pap testreportedlessfear andstress. They confirmed that they had been comfortable during the test and that the test had been painlessand easy to perform. Since these subjects were aware of the importanceandnecessity of the Pap test, they were more willing to have the test in the future (without embarrassment). They were also recommending others to have the test. Similar results were also obtained by Studts et al. [**33**].

Although most of our participants agreed that early diagnosis of cancer increased the treatment success, the participating women also believed that a cancer diagnosis was associated with an early death. In fact, their fear of a positive result sometimes prevented them from having the test. A lack of knowledge about the slow progression of cervical cancer and the necessity of regular screening was hence detected in the studied population. Fear of a cancer diagnosis has been previously introduced as one the most common reasons for not having a Pap test [**28**]. Likewise, in a qualitative study in Malaysia, the women’s wrong beliefs about the purpose of the Pap test (i.e. detecting an existing cervical cancer) and the inevitability of death after a cancer diagnosis were identified as barriers to the participation in cancer screening, detection, and treatment [**26**].

We found that socio-cultural factors play an important role in the Kurdish women’s health. Most participants mentioned their responsibility toward their family as the main reason for taking care of their own health. At the same time, the family’s companionship and encouragement were introduced as important factors in encouragingtheparticipants to have the test. Nevertheless, some women tended to avoid the test despite being supported by their families. Comparable results were indicated byCalvo [**34**]. Conversely, the findings of a study in the United States showed that most Hmong women (younger and older) made decisions about screening independently without counseling their family members, clans, or anyone else [**35**].

Women considered their spiritual beliefs to be able to protect them from serious disease. The Kurdish women’s belief in destiny reduced their perceived individual control over health and disease. This finding was consistent with the findings of Guilfoyle et al. [**20**].

Our participants underscored the importance of mass media as a primary source of health information. Nevertheless, since they mainly used local media, they had never heard of cervical cancer screening programs. Similarly, Panamanian women, who received health information from local media, were found to have limited and sometimes inaccurate information regarding cervical cancer screening [**34**]. However, in Kinshasa, word of mouth communication was the most important source of information [**30**].

In the absence of different sources of health information programs issued by the Ministry of Health and national policies regarding cervical cancer screening, the majority of our participants suggested the role of doctors and healthcare providers as the most important factor in determining a woman’s decision of having a Pap test. A similar finding was indicated by Wong et al. [**26**]. Another study conducted by Horo et al. indicated that only 21.4% of the women performed a cervical cancer screening prior to the study with a delay of several years. Their focus related to no recommendation for cervical cancer screening by doctors and the absence of national policies for the early detection of cervical neoplasia, especially at the beginning age [**36**].

Some participants also highlighted the relations between healthcare providers and women as a major issue in Kurdistan. In other words, healthcare providers were reported not to try to provide women with information about having a Pap test. Poor communication of healthcare personnel has been previously suggested as a barrier to participation in cervical cancer screening programs [**22**]. Similarly, a study in Serbia revealed a poor communication between women and gynecologists and the absence of a proper counseling [**37**]. Consistent findings were also published by Abdullah and Su [**31**] and Ali-Risasi et al. [**30**]. As crucial sources of information, healthcare providers can deliver recommendations and explain the advantages and importance of cervical cancer screening. Impressive communication between healthcare providers and the Kurdish women was identified as the most important factor.

Finally, the weak social influence observed among Kurdish women in the present study can justify the participants’ inappropriate understanding of cervical cancer screening and the lower tendency to have relevant tests. Prior to their own experience, our participants did not hear of the test from any other women. This finding of the present study was in contrast with the results of other studies [**21**,**38**]. 

## Conclusion

Our results underscored the significance of interventions to increase the awareness regarding the importance and necessity of cervical cancer screening among Kurdish women. Providing relevant information can reduce the conflicts, misconceptions, and anxiety among women and eliminate the negative effects of such feelings on women’s decisions to have a Pap test. Since our findings highlighted the absence of a suitable system to encourage women to participate in screening programs in Kurdistan Region of Iraq, policy makers and the Ministry of Healthare required to develop a well-designed program (similar to that implemented in developed countries) to ensure a successful screening. Such a program needs to detect and motivate women who have never had a Pap test.Furthermore, healthcare providers need to acquire better communication skills and recommend Pap tests more often. In addition, Kurdish mass media should be used to educate all women.

**Limitation**

As a qualitative research study, the selection of participants presupposed a small number and these findings might not be generalized to the target population. 

**Acknowledgments**

This study was financially supported by Tehran University of Medical Sciences and Hawler Ministry of Higher Education 

**Conflict of interest**

There is no conflict of interest “Cervical cancer screening by visual inspection in Cˆote d’Ivoire, operational and clinical aspects according to HIV status,”
